# Longitudinally Extensive Transverse Myelitis (LETM) Following Varicella Infection in a 13-Year Immunocompetent Child

**Published:** 2019

**Authors:** Virender KUMAR GEHLAWAT, Jaya SHANKAR KAUSHIK, Poonam MEHTA, Geeta GATHWALA, Rachana DUBEY

**Affiliations:** 1 Department of Pediatrics, Pt B D, Sharma Post Graduate Institute of Medical Sciences, Rohtak, Haryana, India; 2 Department of Pediatrics, Mahatma Gandhi Memorial Medical College, Indore, Madhya Pradesh, India

**Keywords:** Transverse myelitis, Varicella, Chickenpox

## Abstract

Varicella infection in children is a common self-limited illness with neurological complications in less than 0.1% of cases. Longitudinally extensive transverse myelitis (LETM) is uncommon in children following infection with varicella-zoster virus. We describe a case of 13-yr immunocompetent girl with LETM following varicella infection shown a dramatic clinical response to a combination of acyclovir and pulse steroids.

## Introduction

Varicella zoster virus (VZV) infection has been associated with various neurological conditions like acute cerebellar ataxia, aseptic meningitis, encephalitis, stroke, post-herpetic neuralgia, Bell’s palsy, polyradiculoneuropathy and myelitis (1). Immunocompetent children with varicella experience neurologic complications in less than 0.1% (1). Most of these neurological complications including encephalitis and myelitis occur 3-7 d after varicella (1). Longitudinally extensive transverse myelitis (LETM) is a rare complication following varicella infection among immunocompetent children (2, 3). 

We present here a 13-yr girl with LETM following varicella infection who responded clinically to a combination of acyclovir and steroids. 

## Case report

**Figure 1:**
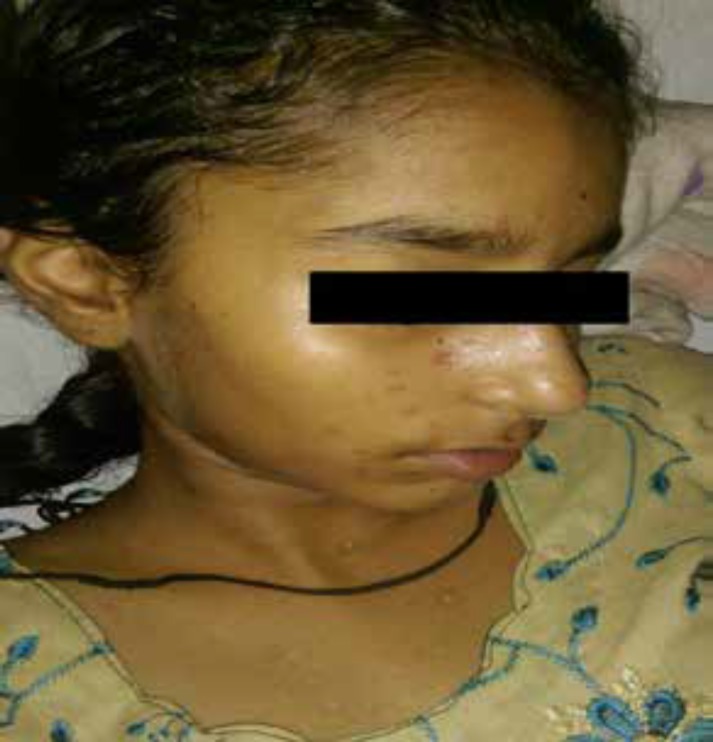
Multiple, small, reddish-brown maculopapular lesions with scab formation and residual hypopigmented lesions over the face and trunk suggestive of varicella skin lesions

A 13-yr girl presented with acute onset weakness in both the lower limbs and upper limb with retention of the bladder for 2 days. Ten days prior to the onset of this illness, she had a history of fever with rash all over the body. Fever had subsided by the 4^th^ day of illness, although the rash evolved from reddish macular to fluid filled pustular lesion leaving behind healed scars all over the body (Figure 1). By the 10^th^ day of illness, she developed sudden onset of non-progressive weakness in both lower limb and upper limb along with bladder retention over 48 hours. There were no areas of sensory loss or paresthesia. There was no history of constipation or bowel involvement. There was no history of blurred vision or pain while moving the eyes. There was no history of preceding trauma, vaccination or drug intake. There was no significant past history of any illness requiring medical attention. There was no contact history of similar rash in her household or school with no other significant family history. She was immunized for age but her vaccination record for varicella was not available. 

On examination, she was conscious alert and oriented. Her heart rate, respiratory rate, and blood pressure were within normal range. Her higher mental functions and cranial nerve examination were unremarkable. Fundus examination was normal with no evidence of papilledema or pappilits. Motor examination revealed hypotonia with symmetrical weakness of lower limbs (Power at least 2/5) and upper limbs (Power at least 4/5). Deep tendon reflexes were brisk with mute plantar response. There were no meningeal signs or cerebellar signs. 

**Figure 2:**
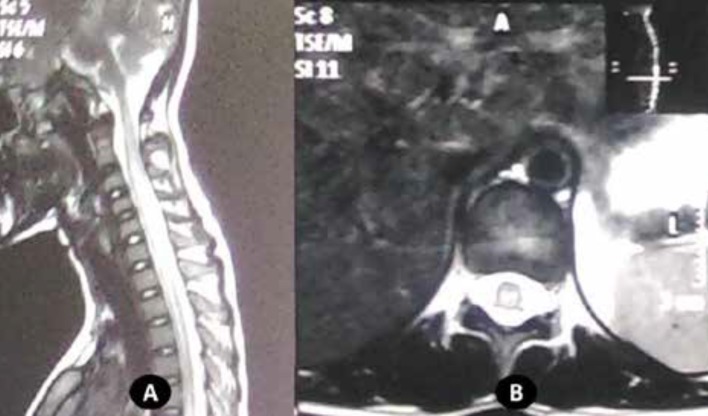
Saggital section (A) and Axial section (B) of MRI spine revealing extensive cord T2 hyperintensity extending from C2 to T1 suggestive of longitudinally extensive transverse myelitis (LETM)

Considering acute onset of upper motor neuron type of symmetric flaccid quadriparesis, neuroimaging of the spine was planned. MRI spine revealed extensive cord T2 hyperintensity with involvement extending from C2 to T1 suggestive of longitudinally extensive transverse myelitis involving cervical spinal cord (Figure 2). MRI Brain along with optic nerve imaging was screened normal. Cerebrospinal fluid (CSF) analysis revealed normal cytology, proteins, and sugars. Serum levels of Aquaporin Aq1b antibody levels were normal. Human Immunodeficiency Virus (HIV) serology was non-reactive. Her antinuclear antibody level (ANA), anti-ds-DNA antibody and angiotensin-converting enzyme (ACE) antibody levels were normal. Anti-ss-A and ss-B level were not performed. 

She was started on intravenous methylprednisolone after ruling out latent tuberculosis along with acyclovir. On treatment, her bladder functions recovered, motor functions had a dramatic improvement and she became ambulatory in the next 2 wk. She was discharged home on oral steroids for 3-4 wk. At 6 wk follow-up, she has no neurological deficits and was attending her schools. At 4 months, follow up her repeat MRI spine was normal. 

## Discussion

Longitudinally extensive transverse myelitis (LETM) refers to intramedullary T2 hyperintensity extending over at least three vertebral bodies (4). There is a wide list of radiological differentials for LETM ranging from infective, inflammatory, demyelinating, vascular and neoplastic causes (4). In our case, the etiology was attributable to varicella infection based on the temporal association of rash with the onset of neurological symptoms and clinical response to a combination of antiviral and immunosuppressant. 

Common neurological complications associated with Varicella Zoster Virus (VZV) include acute cerebellar ataxia, vascular stroke, and encephalitis. Transverse myelitis following varicella infection is uncommon. Most of these cases are reported among elderly and immunocompromised patients (5-9). Atypical neurological manifestations like myelitis are rather more common among immunocompromised patients (7).

Autoimmune etiology for LETM has been largely emerging among those with presumed infectious and parainfectious clinical scenarios. Aquaporin-4 antibody positive neuromyelitis optica following varicella infection has been reported in the literature that responded well to plasma exchange therapy (10). Although Aquaporin 4 antibody titers were negative in our patient, dramatic clinical response to intravenous methylprednisolone raises a possibility of autoimmune-inflammatory pathogenesis to LETM following varicella infection. Other pathogenesis is a direct viral invasion of the spinal cord considered as infective myelitis. Myelitis can follow varicella with latency ranging from 2-6 wk. 

Transverse myelitis may be an isolated entity or may occur in the context of multifocal or even multisystemic disease. In LETM it is often difficult to isolate the virus in cerebrospinal fluid. Demonstration of IgG VZV or VZV DNA in cerebrospinal fluid is variable among those with myelitis following varicella (2,6). Viral PCR and antibody detection were not performed in the case owing to financial and infrastructural constraints. Varicella vaccine is an effective preventive strategy to avert such neurological complications although breakthrough varicella has also been reported to have a similar neurological complication. (2) 

The present case report highlights the case of LETM following varicella infection in an immunocompetent child that responded clinically to a combination of acyclovir and pulse methylprednisolone followed by oral steroids for 3-4 wk. 

**In conclusion, **Transverse myelitis is an uncommon neurological manifestation of varicella infection. The present case report highlights the case of longitudinally extensive transverse myelitis following varicella infection in an immunocompetent child that responded clinically to a combination of acyclovir and pulse methylprednisolone followed by oral steroids for 3-4 weeks. 
